# Diagnostic Accuracy of Contrast-Enhanced Ultrasound Compared with Contrast-Enhanced Computed Tomography in the Follow-Up of Hepatocellular Carcinoma Treated with Radiofrequency Ablation

**DOI:** 10.3390/cancers17172808

**Published:** 2025-08-28

**Authors:** Giulia Gori, Stefano Mazza, Carlo Ciccioli, Erica Bartolotta, Daniele Alfieri, Francesca Torello Viera, Aurelio Mauro, Davide Scalvini, Letizia Veronese, Chiara Barteselli, Carmelo Sgarlata, Marco Bardone, Laura Rovedatti, Simona Agazzi, Elena Strada, Lodovica Pozzi, Alessandro Vanoli, Chandra Bortolotto, Antonio Facciorusso, Antonio Di Sabatino, Valentina Ravetta, Andrea Anderloni

**Affiliations:** 1 Department of Internal Medicine and Medical Therapeutics, University of Pavia, 27100 Pavia, Italy; 2 Gastroenterology and Endoscopy Unit, Fondazione IRCCS Policlinico San Matteo, 27100 Pavia, Italy; 3 Section of Gastroenterology and Hepatology, Dipartimento Di Promozione Della Salute, Materno Infantile, Medicina Interna e Specialistica Di Eccellenza (PROMISE), University of Palermo, 90127 Palermo, Italy; 4 Unit of Anatomic Pathology, Department of Molecular Medicine, University of Pavia, 27100 Pavia, Italy; 5 Diagnostic Imaging and Radiotherapy Unit, Department of Clinical, Surgical, Diagnostic and Pediatric Sciences, University of Pavia, 27100 Pavia, Italy; 6 Radiology Institute, Fondazione IRCCS Policlinico San Matteo, 27100 Pavia, Italy; 7 Gastroenterology Unit, Department of Experimental Medicine, University of Salento, 73100 Lecce, Italy; 8 Internal Medicine Unit, Fondazione IRCCS Policlinico San Matteo, 27100 Pavia, Italy

**Keywords:** hepatocellular carcinoma, contrast-enhanced computed tomography, contrast-enhanced ultrasound, radiofrequency ablation

## Abstract

Background: Contrast-enhanced computed tomography (CECT) is considered the gold standard for assessing therapeutic response in hepatocellular carcinoma (HCC) following locoregional treatments. More recently, contrast-enhanced ultrasound (CEUS) has emerged as a promising, cost-effective alternative, although evidence supporting its use in this context remains limited. This study aimed to evaluate the diagnostic performance of CEUS compared to CECT in assessing local response of HCC nodules treated with radiofrequency ablation (RFA). Methods: We retrospectively analyzed a consecutively enrolled cohort of patients undergoing RFA for HCC at IRCCS San Matteo Hospital, Pavia, between January 2017 and January 2022. Follow-up imaging included both CEUS and CECT at predefined time points. Imaging follow-up, MRI, and/or histological results were used as the reference standard to compare CEUS and CECT. Results: A total of 55 patients (mean age 74 years, 64% male) with 79 HCC nodules were included. Complete response was observed in 57 nodules (72%), all correctly identified by CEUS, whereas 4 cases were misclassified by CECT. Among the 22 nodules (28%) with residual disease, CEUS correctly identified 15 cases, while 17 were detected by CECT; notably, CEUS identified 4 lesions missed by CECT, which identified 6 missed at CEUS. Combined, the two modalities detected 21 (96%) out of 22 residual tumors. CEUS showed a sensitivity of 68.1%, specificity of 100%, and diagnostic accuracy of 91.1%, compared to 68.0%, 98.1%, and 88.6%, respectively, for CECT, with no significant differences. Conclusions: CEUS is a reliable, non-inferior, and complementary modality to CECT for evaluating treatment response of HCC following RFA. Larger prospective studies are warranted to further define its role in clinical practice.

## Simple Summary

Contrast-enhanced ultrasound (CEUS) is an affordable and effective imaging modality for assessing the treatment response of hepatocellular carcinoma (HCC) nodules following locoregional therapies. However, data on its use in this specific setting remain limited, and contrast-enhanced computed tomography (CECT) is still considered the gold standard. In this study, we compared CEUS and CECT in evaluating HCC response after radiofrequency ablation (RFA). We observed a very good agreement between the two techniques, with no significant differences in sensitivity, specificity, or diagnostic accuracy. Notably, the combined use of CEUS and CECT improved the detection rate of residual disease. Our findings support CEUS as a reliable and non-inferior tool to CECT for post-RFA assessment of HCC nodules.

## 1. Introduction

Hepatocellular carcinoma (HCC) is the most common primary liver cancer and ranks sixth among global cancers [1,2]. Over 80% of HCC cases occur in patients with liver cirrhosis, regardless of underlying cause, which represents the terminal stage of many chronic inflammatory liver diseases [3]. The main risk factors for HCC are hepatitis C virus (HCV) and/or hepatitis B virus (HBV) infection, alcohol consumption, aflatoxin exposure, and steatohepatitis within the context of metabolic syndrome [4].

HCC is one of the few malignancies that can be diagnosed non-invasively based solely on imaging criteria. According to international guidelines, the diagnosis of HCC in at-risk patients relies on identifying a characteristic vascular pattern: arterial phase hyperenhancement followed by washout in the portal venous or delayed phases. Both the European Study of Liver Diseases (EASL) and the American Association for the Study of Liver Diseases (AASLD) guidelines recommend that, in patients with chronic hepatitis B or cirrhosis, nodules larger than 10 mm exhibiting this typical vascular profile on at least one imaging modality (MRI, CT, or CEUS) can be diagnosed as HCC non-invasively [5,6]. CEUS has emerged as a valuable tool in both diagnosis and follow-up. It offers several advantages, including the absence of ionizing radiation, excellent temporal resolution, and the use of safe, purely intravascular contrast agents. Unlike CT and MRI contrast agents, CEUS agents do not carry nephrotoxic risk, making the technique suitable for patients with renal impairment. Additionally, CEUS allows real-time assessment of vascular dynamics, which can be particularly helpful in identifying early washout or subtle perfusion changes [7].

The Barcelona Clinic Liver Cancer (BCLC) classification, updated in 2022, stratifies HCC into five stages and guides treatment selection based on tumor burden, liver function, and performance status [8]. Curative therapies—including hepatic resection, liver transplantation, and percutaneous ablation—are indicated for very early- (BCLC-0) and early-stage (BCLC-A) diseases, defined by preserved liver function, good performance status, and limited tumor size and number [8,9]. While resection remains the first-line option in eligible patients, ablation is increasingly adopted in those deemed inoperable, particularly for nodules ≤ 2 cm [10]. Ablation thus represents a key modality within the curative-intent strategy for early-stage HCC.

At present, contrast-enhanced CT (CECT) or contrast-enhanced MRI (CEMRI) represents the reference standard for evaluating residual disease following locoregional treatment. According to European guidelines, CEUS is recommended for post-treatment evaluation in cases where CECT or CEMRI are inconclusive or contraindicated. Furthermore, CEUS may serve as a tool for secondary surveillance, typically performed every 3–4 months, in conjunction with periodic CECT or CEMRI. This approach aims to facilitate the early detection of recurrence while minimizing cumulative contrast exposure and radiation dose [11].

Among locoregional treatment strategies, radiofrequency ablation (RFA) remains the most widely applied, with reported success rates ranging from 75% to 94%, particularly for lesions smaller than 2–3 cm [12,13]. Alternative ablation techniques such as microwave thermal ablation (MWTA) offer greater energy delivery, enabling treatment of tumors > 3 cm with shorter application times. MWTA achieves higher intra-tumoral temperatures and larger ablation volumes than RFA but carries a higher risk of complications [14]. Another option is percutaneous ethanol injection (PEI), a low-cost method now reserved for small lesions when RFA is not feasible. Although once widely used, PEI has been largely supplanted by RFA, as meta-analyses have demonstrated RFA superiority in overall survival and local control [15,16].

Accurate post-treatment imaging is essential to evaluate therapeutic response and detect residual disease. CECT and CEMRI remain the reference standards for post-ablation assessment, but CEUS is increasingly being used as a complementary tool, especially given its safety profile, accessibility, and ability to provide real-time dynamic vascular assessment. However, current evidence supporting CEUS as a standalone modality in post-treatment response assessment is still limited [17,18,19]. Most studies suggest that its diagnostic accuracy may be comparable to that of CECT when used under optimal conditions and by experienced operators. CEUS is particularly useful in secondary surveillance or when CT/MRI findings are equivocal or contraindicated.

To improve diagnostic standardization, the American College of Radiology firstly introduced the Liver Imaging Reporting and Data System (LI-RADS) Treatment Response (LR-TR) algorithm in 2017. This algorithm represents an expansion of the LI-RADS system firstly introduced in 2011 [20], and was developed to standardize CECT/CEMRI-based assessment of HCC local response after locoregional therapies, including RFA [21]. In the last LI-RADS update in 2024, CEUS was also included in the treatment response algorithm [22].

This study investigates the diagnostic performance of CEUS compared with CECT for assessing HCC response after RFA, evaluating its potential as a reliable alternative in specialized hepatology centers.

## 2. Materials and Methods

### 2.1. Study Design and Outcomes Definition

For this retrospective, single-center study, all patients diagnosed with HCC undergoing HCC treatment by RFA between January 2017 and January 2022 were consecutively enrolled. At enrolment, the following data were collected: age, gender, date of diagnosis, date of procedure, date of radiological follow-up examinations (CEUS and CECT), etiology of liver disease, Child-Pugh score, nodule size, and HCC grade on histological examination when available. We included only patients treated with the same therapeutic approach (i.e., RFA) in order to obtain a homogeneous cohort, thereby avoiding biases related to different therapies. Therefore, all patients included in the study were classified as BCLC 0 (very early) or BCLC A (early) stage, to be suitable for locoregional curative treatment.

Further exclusion criteria were as follows: ≥3 previous treatments, prior transarterial chemoembolization (TACE), <18 years old age, pregnancy.

According to institutional protocols, RFA was performed within one month of HCC diagnosis. First follow-up imaging with both CEUS and CECT was performed at one month post-treatment to assess therapeutic response. Local treatment response was defined according to LI-TR criteria as nonviable, equivocal, or viable.

**Figure 1:**
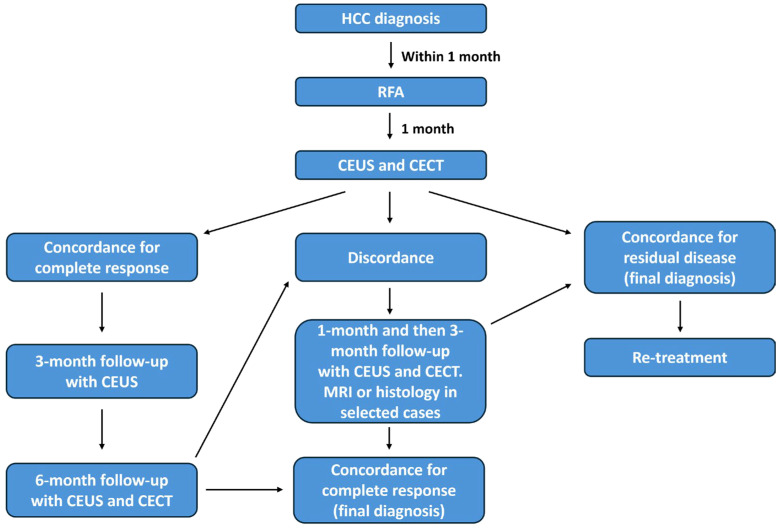
Flowchart illustrating the clinical management of the study cohort, from HCC diagnosis to post-RFA follow-up. Possible scenarios based on CEUS and CECT findings are shown.

Complete response (CR) and residual disease (RD) were considered as final outcomes for RFA-treated HCC. For this purpose, a composite reference standard considering either the concordance between CEUS and CECT and the post-RFA follow-up was assumed. Specifically, the following scenarios were considered (Figure 1):

CR at both CEUS and CECT → 3-month follow-up with CEUS and then 6-month follow-up with CEUS and CECT → concordance → final diagnosis of CR.

RD at both CEUS and CECT → final diagnosis of RD → management of residual lesion.

CEUS/CECT discordance → 1-month follow-up with both techniques and then every 3 months, until a final diagnostic agreement was reached. In selected cases, MRI or histological examination were used to assess the response.

The study was approved by the local Ethics Committee (number 0004836/25) and was performed according to the Helsinki declaration. All patients gave their informed consent for the procedures.

### 2.2. Post-RFA Assessment of HCC Module Response

CEUS was performed using a Hitachi Aloka ultrasound machine (Prosound, F75, Arietta V70) (Hitachi Aloka Medical, Ltd., Tokyo, Japan) equipped with multifrequency convex transducers (1.0–5.0 MHz). SonoVue (Bracco, Milan, Italy) was used as contrast agent, which is composed of sulfur hexafluoride microbubbles (8 µL/mL), prepared by reconstituting 25 mg of lyophilized powder in 5.0 mL of 0.9% sodium chloride solution; the contrast was administered as a 2.4 mL bolus through a 19G cannula in an antecubital vein, followed by a 5.0 mL saline flush. The ultrasound systems were optimized for CEUS, using a mechanical index (MI) of ≤0.04 to minimize microbubble disruption. A timer on-screen enabled accurate assessment of enhancement phases. According to the 2020 European guidelines [11], the first 60–90 s post-injection were continuously observed to evaluate the arterial and portal phases, while intermittent imaging was used for the late phase. Recordings documented the three contrast-enhancement phases: the arterial phase, which begins approximately 10–20 s after contrast injection and ends at 25–35 s; the portal-venous phase, which spans from about 30–45 s to 120 s; and the late phase, which begins beyond 120 s, with contrast washout typically occurring between 240 and 360 s. In cirrhotic livers, the typical CEUS enhancement pattern of HCC includes arterial hyperenhancement (hyperechoic), followed by an isoechoic appearance during the portal-venous phase, and isoechoic to hypoechoic washout in the late phase. Each treated nodule was evaluated independently by two internal medicine physicians with over 10 years of experience in liver ultrasound and HCC management.

Triple-phase CECT was performed using a multidetector CT scanner, acquiring images during arterial (early and late), portal-venous, and delayed phases in a single breath-hold. The contrast agent, with vasculo-interstitial distribution, rapidly transitions from the vascular to the extracellular compartment, allowing evaluation of neoplastic enhancement and washout characteristics. Typically, HCC lesions appear hypodense on baseline CT, show rapid contrast uptake in the arterial phase, and demonstrate washout during portal-venous and delayed phases.

Both examinations were performed by two operators with more than 10 years of experience in liver imaging. CEUS and CECT images were interpreted independently and in a blinded manner (i.e., each radiologist was unaware of the findings from the other imaging modality) to reduce potential diagnostic bias. In case of discordance, images were reviewed by a third expert operator and discrepancies were resolved by consensus discussion.

**Figure 2:**
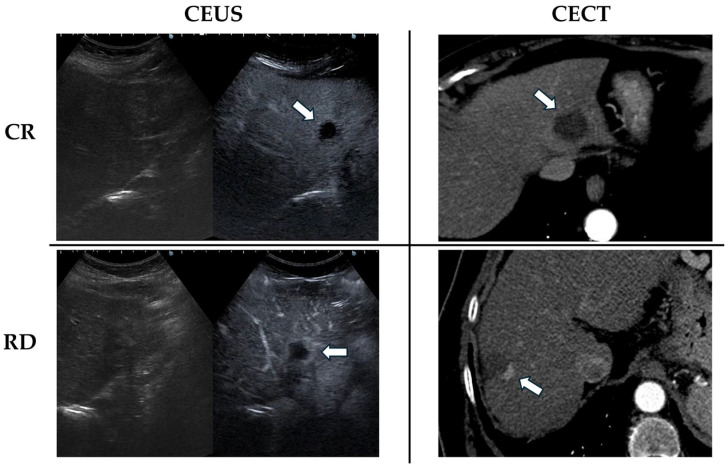
Illustrative images of CR and RD appearance at both CEUS and CECT. Treated lesions are indicated by white arrows. CR appears on CT as a low-density lesion with adequate margins, and on CEUS as an avascular area without peripheral enhancement. In case of RD, an avascular area with a peripheral hyperenhancing viable tissue is clearly visible at both CEUS and CECT.

A representative image of CR and RD at both CEUS and CECT is shown in Figure 2.

### 2.3. Aims

The primary objective was to evaluate the diagnostic reliability of CEUS in assessing the therapeutic response of HCC nodules treated with RFA, distinguishing between RD and CR.

Secondary objectives were as follows: to compare CEUS and CECT in the assessment of HCC nodule response after RFA treatment; to assess the efficacy of RFA performed in a tertiary care hospital within an interventional ultrasound unit specialized in chronic liver disease and HCC management; to explore whether patient-related variables (age, sex, liver disease etiology) and nodule-related characteristics (diameter, histological grade, morphology) correlate with treatment outcome.

### 2.4. Statistical Analysis

Categorical variables were described as absolute frequency and percentage. Continuous variables with normal distribution were described as mean ± standard deviation (SD), whereas the continuous variables without normal distribution were given as median and range. The diagnostic performance of the two methods, CEUS and CT, in identifying RD as positive result will be assessed by calculating the following: sensitivity, defined as true positives (TP) divided by the sum of TP and false negatives (FN); specificity, defined as true negatives (TN) divided by the sum of TN and false positives (FP); and diagnostic accuracy, defined as the sum of TP and TN divided by the sum of TP, FP, FN, and TN. All values will be given with relative 95% confidence interval (95%CI). The agreement between the two different radiological methods in the allocation of treated HCC nodules will be established using Cohen’s kappa coefficient. Specifically, levels of agreement will be classified as poor (k < 0.20), fair (k > 0.20 to <0.40), moderately good (k > 0.40 to <0.60), good (k > 0.60 to <0.80), or very good (k > 0.80 to 1.00). The efficacy of RFA in the treatment of HCC nodules will be calculated based on the number of successfully treated cases and the number of cases with residual disease related to all treated cases. The correlation between baseline parameters and RFA efficacy will be established through logistic regression analysis, and the results will be expressed as hazard ratio (HR) and 95% confidence interval. Statistical significance will be set at a *p* value < 0.05. All analyses will be performed using IBM SPSS Statistics (release 25; IBM Corporation, Armonk, NY, USA).

## 3. Results

Fifty-five patients with a new diagnosis of HCC subsequently treated with RFA were included. The analyzed population had a mean age of 74.3 years old and consisted of 20 female patients (36.4%). All patients had chronic liver disease, with a clear prevalence of viral etiology (63.6%), predominantly HCV (52.8%). Demographic and baseline clinical features are reported in Table 1. Median follow-up time was 24 months (range 8–46).

A total of 79 nodules were treated, with cases of treatments for different nodules on the same patient over the years (Table 1). The mean diameter of the nodules was 23 mm. 53 of these nodules (67%) were in the right lobe (V 21.5%, VI 8.9%, VII 15.1%, VIII 24.1%), and the remaining ones in the left lobe (II 8.9%, III 5.1%, IV 16.4). Echogenicity of the nodules was predominantly inhomogeneous (46.8%), followed by hypoechoic (32.9%) and hyperechoic (15.2%) patterns, with only a small proportion being isoechoic (2.5%). Biopsy was performed in 50.5% of patients: nodules showed histology G1 in 25.3%, G2 in 21.3%, and G3 in 3.7% (Table 2).

Of the 79 HCC nodules treated with RFA, 57 (72%) showed a complete response, while a residual disease was identified in 22 cases (28%) (Table 2).

At the 1-month follow-up, CEUS correctly identified all cases of CR (57/57), while CECT detected 53 (93%). In 3 cases, CECT provided equivocal results, while 1 case was erroneously classified as RD (Table 3).

Regarding the 22 RD cases, CEUS correctly identified 15 cases (68%) compared to the 17 (77%) correctly discriminated by CECT. Of the 7 cases misdiagnosed at CEUS, one was classified as equivocal. Notably, CEUS correctly identified 4 cases of RD that CECT failed to diagnose, while CECT recognized 6 residual lesions not visualized by CEUS. Concordant identification of RD by both techniques occurred in 12 cases (55%). One case of RD was missed by both imaging modalities at the first follow-up and was subsequently identified at the 6-month follow-up. This lesion was a hypoechoic 18 mm nodule located in liver segment IV, with first follow-up imaging performed at 1 month post-RFA. The combined methods successfully identified 21 out of 22 cases (95.5%) of RD (Table 3).

**Figure 3:**
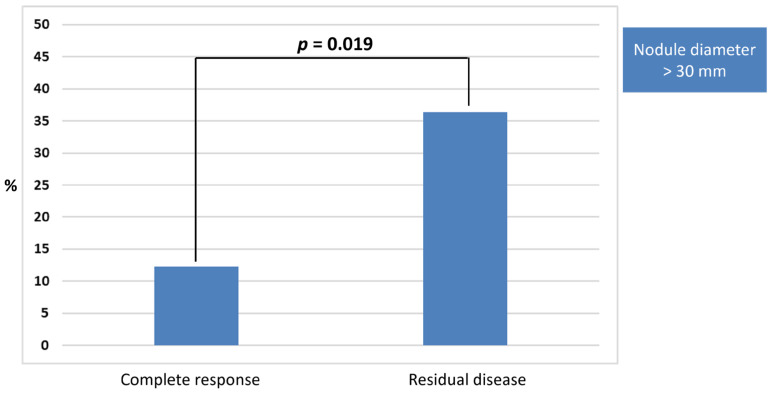
Histogram comparing the frequency of HCC nodule diameter > 30 mm in patients experiencing complete response vs. residual disease after RFA treatment. A significant correlation was found at the logistic regression analysis.

No statistically significant correlations were identified between treatment efficacy and baselines variables related to the study population (age, sex, etiology of liver disease, Child-Pugh score). Among nodules features, a nodule diameter > 30 mm was correlated with a reduced treatment efficacy at logistic regression analysis (46.7% for nodule > 30 mm vs. 78.1% for nodule < 30; HR 0.25 95%CI 0.76–0.79; *p* = 0.019) (Figure 3).

CEUS showed a sensitivity of 68.2% (95%CI 45.13–86.14%), specificity of 100% (95%CI 93.73–100%), and accuracy of 91.14% (95%CI 82.59–96.36%), compared to 77.3% (95%CI 45.6–85.05%), 93% (95%CI 90.11–99.95%), and 88.6% (95%CI 79.47–94.66%), respectively, for CECT (Table 4). These differences were not statistically significant (*p* > 0.05). Considering the association between the two techniques, a sensitivity of 95.5%, specificity of 100%, and an accuracy of 98.7% were reached.

The agreement between CEUS and CECT was very good, as indicated by a Cohen’s kappa of 0.82 (95%CI 0.78–0.87).

## 4. Discussion

In this retrospective analysis, we evaluated the diagnostic performance of contrast-enhanced ultrasound (CEUS) compared to contrast-enhanced computed tomography (CECT) in assessing treatment response following radiofrequency ablation (RFA) in patients with hepatocellular carcinoma (HCC). HCC locoregional therapies, originally conceived for palliative purposes, as bridging tools to liver transplantation, or as a means of tumor downstaging, have evolved into curative treatment options. These approaches are now considered valid alternatives to partial hepatectomy or liver transplantation, particularly in the treatment of small HCCs. This is largely due to the typically intrahepatic nature of HCC, which tends to remain localized for extended periods before developing extrahepatic spread. Among these, RFA has demonstrated the highest therapeutic efficacy. Nonetheless, the significant risk of local recurrence following ablative therapies underscores the importance of rigorous post-treatment surveillance, with a focus on radiological evaluation of treatment response.

In our retrospective study, we analyzed 55 patients with a total of 79 nodules treated with RFA over a five-year period. The overall complete response (CR) rate was 72%, consistent with previous data reporting CR rates after RFA ranging from 70% to 94% [23,24,25].

In our real-life cohort, several factors may have reduced the RFA efficacy. First, 67% of nodules were located in the right hepatic lobe, particularly in segments VIII and V—areas known to be technically challenging for ablation and prone to diagnostic uncertainty. Moreover, the mean tumor size at diagnosis was 23 mm, indicating that most nodules were beyond the early stage (typically < 2 cm), and were thus associated with a higher risk of local recurrence [6].

At the 1-month follow-up, CEUS identified all complete responses, outperforming CECT, which missed four cases including one false positive, potentially leading to unnecessary retreatment; this discordance was solved by MRI execution, which showed CR, subsequently confirmed by follow-up. These results suggest that CEUS reliably detects complete response to treatment, supporting its role in post-RFA surveillance.

CEUS detected 68% of residual disease (RD) cases, only slightly lower than CECT (77%), with the two techniques reaching agreement in 55% of cases. Specifically, CEUS failed to detect 7 out of 22 RD cases (including one equivocal case), suggesting that vascular patterns in residual tumor may be subtle or atypical at early follow-up. Importantly, CEUS allowed correct allocation of 80% of RD cases missed by CT, while CECT correctly identified 86% of RD cases not detected by CEUS. This might be explained considering that CEUS benefits from continuous vascular phase examination, which improves detection of small peripheral foci, while CECT may perform better for deeply located lesions or when acoustic windows are limited.

Overall, the combined use of CEUS and CT correctly identified 96% of cases. Only one treated nodule was misdiagnosed by both methods due to periablational hyperemia, with recurrence only recognized months later, following resolution of the reactive halo, leading to repeat RFA.

Our results demonstrate that CEUS provides high specificity (100%) and overall diagnostic accuracy (91.1%), comparable to those obtained with CECT, which showed 93% specificity and 98% accuracy in the same cohort. Although not statistically significant, specificity of CEUS was higher compared to CECT. This result highlights a potential advantage of CEUS: its ability to provide real-time dynamic imaging, which minimizes the risk of missing brief arterial phase enhancements and allows prolonged observation to detect late-onset washout, thereby reducing the likelihood of false-negative results.

The moderate sensitivity of CEUS for detecting RD (68.2%) observed in our cohort is in line with findings from other studies that emphasize its operator dependence and limitations in deeply located or small residual lesions [7,26]. Anyway, CEUS sensitivity was not significantly different from that of CECT (77%). A previous study comparing CEUS with CT/MRI in the monitoring of early intrahepatic recurrences of HCC after curative treatment with resection or RFA reported a CEUS sensitivity of 97% and a specificity of 68%, whereas CT/MRI showed corresponding values of 71.7% and 72%, respectively. These findings suggest that CEUS can be an effective imaging modality for post-treatment HCC monitoring [27]. In another study comparing CEUS and MRI, no substantial differences were observed in terms of sensitivity (71.0–85.0% for CEUS and 80.9–92.0% for MRI), specificity (97.4–99.2% vs. 98.5–99.9%), and area under the ROC curve (0.85–0.92 vs. 0.91–0.96) when used as part of secondary surveillance algorithms in a single-examination setting. However, MRI was associated with a higher number of equivocal findings, often requiring subsequent CEUS assessment. CEUS was able to resolve all of these equivocal cases, which typically stemmed from difficulties in distinguishing residual disease from vascular or shunting alterations commonly seen in early post-treatment phases [28]. Overall, this evidence supports the non-inferiority of CEUS in the post-RFA assessment of HCC nodules and may have important clinical implications given its wide availability, low cost, radiation-free nature, and favorable safety profile. Patients with renal impairment or iodine allergy may therefore be considered excellent candidates for CEUS. On the other hand, operator-dependent nature of CEUS must be considered, and further evidence is warranted to define its role in clinical practice.

Notably, the combined use of CEUS and CECT achieved a sensitivity of 95.5% and an accuracy of 98.7%, highlighting the complementary role of the two techniques in detecting RD. We may therefore assume that in cases of doubtful results at either CECT or CEUS, the second technique might be performed to reach a definitive conclusion regarding the presence of RD. Furthermore, the diagnostic agreement between CEUS and CECT was excellent (Cohen’s kappa = 0.82), supporting CEUS as a reliable adjunct in specialized hepatology settings.

Importantly, we identified that larger nodule size (>30 mm) was associated with reduced treatment efficacy (*p* = 0.019), in line with previous studies reporting that lesion size and tumor biology influence the rate of local recurrence after RFA [23,29,30,31].

To ensure optimal patient outcomes, it is essential to assess the effectiveness of locoregional treatment by establishing an appropriate imaging follow-up [32]. The ability to differentiate viable from non-viable tumor tissue is critical in guiding timely therapeutic decisions and optimizing patient management. in the surveillance and follow-up of treated HCC. CEUS offers significant advantages, including reduced exam time, lower costs, and minimal patient toxicity. Beyond initial diagnosis, it aids in treatment planning and real-time guidance during procedures and proves reliable in monitoring treatment response by accurately detecting residual viable tumor tissue. However, the exclusive use of CEUS for post-treatment evaluation, thus without complementary imaging with CT or MRI, remains uncommon in clinical practice. In most cases, patients undergo multiple imaging modalities, even when the added value of cross-sectional techniques appears limited.

This study includes patients monitored since 2017, the year in which the CEUS LI-RADS guidelines were introduced [33], providing standardized criteria for interpreting enhancement patterns and contrast kinetics in the characterization of HCC, including standardized approach for evaluating treated hepatic lesions, known as LI-RADS Treatment Response (LR-TR). This system classifies lesions as nonviable, equivocal, or viable based on imaging features [34]. The algorithm has been successfully validated for use with CT and MRI, while a dedicated CEUS-based version has only recently been released [35]. A 2019 study demonstrated that the LR-TR algorithm has a high predictive value for assessing tumor viability following locoregional treatment [36]. According to the 2024 CEUS TRA version the evaluation is based on both intralesional and perilesional contrast enhancement patterns. The algorithm adopts broader criteria for intralesional enhancement to increase sensitivity for detecting tumor viability, while applying stricter criteria to perilesional enhancement in order to avoid misclassifying post-treatment reactive changes as viable tumor. In the subsequent assessment phase, intralesional and perilesional findings are integrated to assign a single LR-TR category: LR-TR nonviable, LR-TR equivocal, or LR-TR viable. These refinements suggest that CEUS now holds potential comparability with second-level imaging modalities such as CT and MRI for post-treatment HCC evaluation.

This study presents several limitations. First, the retrospective and monocentric nature of the study, with a relatively limited number of patients and nodules, which may affect the generalizability of the findings. Second, although a composite reference standard was used (including imaging follow-up, CEMRI, and histology), histological confirmation was not available in most cases, potentially affecting diagnostic certainty. Third, the timing between CEUS and CECT, although brief, was not standardized, which could introduce temporal bias in lesion appearance. Finally, as all CEUS procedures were performed by experienced operators in a high-volume center, the diagnostic performance reported here may not be reproducible in lower-expertise settings.

Strengths of the study are the homogeneity of the study cohort, with particular reference to the regularity and adherence to the follow-up after HCC treatment, and the high reliability of expert operators performing imaging procedures; the real-life cohort, which gives a reliable picture of what we observe in clinical practice; the long-term median follow-up that make the outcomes and results more robust.

## 5. Conclusions

In conclusion, our study confirms that CEUS is a highly specific and accurate modality for evaluating local response of HCC nodules treated with RFA, with diagnostic performance comparable to CECT in experienced hands. Given its favorable safety profile and real-time imaging capabilities, CEUS may serve as a reliable alternative, particularly in patients with renal dysfunction or contraindications to iodinated contrast agents. Moreover, the combined use of CEUS and CECT can maximize diagnostic yield, particularly in equivocal and challenging cases, and may represent a cost-effective, low-risk approach to post-treatment monitoring. Further prospective, multicenter studies are warranted to validate our findings and to define the role of CEUS in the follow-up of HCC treated with locoregional therapies in routine clinical practice.

## Figures and Tables

**Table 1:** Demographic and baseline clinical characteristics of the 55 patients analyzed in the study.

Patients’ Characteristics	Total *n* = 55
Age at enrollment, years, median (SD)	74.3 (6.3)
Sex (F), *n* (%)	20 (36.4)
Etiology:	
HCV	29 (52.8)
NASH	7 (12.7)
Alcohol	7 (12.7)
HBV	6 (10.9)
Cryptogenic	6 (10.9)
Child-Pugh score, *n* (%)	
A	46 (83.6)
B	9 (16.4)
BCLC stage	
0 (Very early)	12 (21.8)
A (Early)	43 (78.2)
Number of HCC nodules, *n* (%)	
1	35 (63.6)
2	16 (29.1)
3	4 (7.3)
Time from RFA to CECT, days, median (range)	40 (9–95)
Time from RFA to CEUS, days, median (range)	43 (10–97)
Follow-up time, median, range	24 (8–46)

CECT: contrast-enhanced computed tomography; CEUS: contrast-enhanced ultrasound; HBV: hepatitis B virus; HCV: hepatitis C virus; NASH: nonalcoholic steatohepatitis; RFA: radiofrequency ablation; SD: standard deviation.

**Table 2:** Main characteristics of the 79 patients’ nodules analyzed in the study.

Nodules Characteristics	Total *n* = 79
Diameter, mm, median (range)	23 (10–58)
Hepatic segment location, *n* (%)	
II	7 (8.9)
III	4 (5.1)
IV	13 (16.4)
V	17 (21.5)
VI	7 (8.9)
VII	12 (15.1)
VIII	19 (24.1)
Echogenicity, *n* (%)	
Inhomogeneous	38 (48.1)
Hypoechoic	26 (32.9)
Hyperechoic	12 (15.2)
Isoechoic	2 (2.5)
Grading (*n* = 40), *n* (%)	
G1	17 (42.5)
G2	20 (50)
G3	3 (7.5)
Local response after RFA, *n* (%)	
Complete response	57 (72.1)
Residual disease	22 (27.9)

RFA: radiofrequency ablation.

**Table 3:** Assessment of local response at 1-month follow-up after RFA based on CEUS and CECT.

			Final Outcome	
Technique	Result	Tot (*n* = 79)	CR (*n* = 57)	RD (*n* = 22)
CEUS	Nonviable	63	57	6
	Viable	15	0	15
	Equivocal	1	0	1
CECT	Nonviable	56	53	3
	Viable	18	1	17
	Equivocal	4	3	1
CEUS + CECT	Nonviable	58	57	1
	Viable	21	0	21
	Equivocal	0	0	0

CEUS: contrast-enhanced ultrasound; CECT: contrast-enhanced computed tomography; CR: complete response; RD: residual disease.

**Table 4:** Sensitivity, specificity and diagnostic accuracy of CEUS and CECT in the assessment of local response at 1-month follow-up after RFA.

Technique	Sensitivity	Specificity	Accuracy
CEUS	68.2%	100%	91.1%
CECT	77.3%	93%	88.6%
CEUS + CECT	95.5%	100%	98.7%

CEUS: contrast-enhanced ultrasound; CECT: contrast-enhanced computed tomography.

## Data Availability

The research data are stored in an institutional repository and will be shared upon request to the corresponding author.
